# Immunotherapy rechallenge in gastric cancer: resistance mechanisms, molecular stratification, and precision decision-making

**DOI:** 10.3389/fimmu.2026.1820652

**Published:** 2026-05-20

**Authors:** Yu-cai Jiang, Lin-lin Zheng, Ming-gui Fu, Bi-jin Zheng

**Affiliations:** 1 Department of Pharmacy, Affiliated Hospital of Putian University, Putian, Fujian, China; 2 Department of Oncology, Affiliated Hospital of Putian University, Putian, Fujian, China; 3 School of Pharmacy and Medical Technology, Putian University, Putian, Fujian, China

**Keywords:** gastric cancer, immune checkpoint inhibitor, immunotherapy, molecular stratification, precision therapy, rechallenge, resistance mechanisms

## Abstract

**Background/objectives:**

Immune checkpoint inhibitors (ICIs) have changed the treatment landscape of advanced gastric cancer (GC). However, acquired resistance remains common. For patients who initially benefit and later progress, immunotherapy rechallenge is biologically plausible but still investigational.

**Approach:**

This narrative review synthesizes mechanistic, translational, biomarker, and clinical evidence on ICI resistance and rechallenge in GC.

**Results:**

Resistance may involve tumor-intrinsic plasticity, hypoxia- and metabolism-driven remodeling, impaired antigen presentation, suppressive tumor microenvironment (TME) components, T-cell exhaustion, and compensatory checkpoints such as Lymphocyte-activation gene 3 (LAG-3), T-cell immunoglobulin and mucin-domain containing-3 (TIM-3), T-cell immunoreceptor with Ig and ITIM domains(TIGIT), and V-domain Ig suppressor of T cell activation (VISTA). Epstein-Barr virus (EBV)-positive and Microsatellite instability-high (MSI-H)/Deficient mismatch repair (dMMR) tumors often show immune-inflamed features, but they may still develop HLA class I loss, immune editing, or cold-tumor phenotypes. Direct GC-specific rechallenge evidence remains limited and is mainly retrospective or case-series based. Therefore, mechanistic and non-GC data should be interpreted as indirect and hypothesis-generating.

**Conclusions:**

ICI rechallenge in GC should be regarded as a biomarker-informed investigational strategy rather than a standard of care. Prospective trials, standardized definitions, validated biomarkers, and careful toxicity monitoring are needed.

## 1 Introduction

### 1.1 Current status and clinical challenges of immunotherapy for gastric cancer

ICIs have reshaped the treatment of advanced gastric cancer. Several ICIs are now used in selected clinical settings, usually as part of ICI-based combinations or biomarker-defined strategies. Despite this progress, ICI monotherapy has modest activity in unselected populations, immune-related adverse events remain clinically relevant, and validated predictive biomarkers are still limited mainly to MSI-H/dMMR status and selected Programmed Death-Ligand 1(PD-L1)-defined contexts (1–4). Acquired resistance after an initial response is therefore a major barrier to durable benefit.

### 1.2 Clinical needs and scientific significance of rechallenge with immunotherapy

As first-line ICI-based therapy expands, more patients will progress after prior Programmed Death-1(PD-1)/PD-L1 exposure. The key clinical question is whether selected patients should receive ICI continuation, readministration, or true rechallenge. Current GC-specific evidence is limited, heterogeneous, and mostly retrospective. Therefore, rechallenge should be framed as investigational and patient-selective. It may be biologically plausible in settings such as oligoprogression, durable prior benefit, or targetable adaptive resistance, but prospective validation is required before routine use.

### 1.3 Framework of this review

This article is a narrative, non-systematic review. It does not include protocol registration, duplicate screening, a PRISMA flow diagram, quantitative synthesis, or formal risk-of-bias scoring. Instead, it provides a critical synthesis of representative mechanistic, translational, biomarker, and clinical studies relevant to ICI resistance and rechallenge in gastric cancer.

Additional methodological clarification: To address the risk of being interpreted as a systematic review, we have removed any implication that a PRISMA-style systematic review was performed. No formal protocol registration, duplicate screening, exhaustive database search, quantitative synthesis, or formal risk-of-bias scoring was undertaken.

Literature handling followed a transparent narrative process: (i) scoping searches were used to identify representative mechanistic, translational, biomarker, and clinical studies relevant to ICI resistance and rechallenge in gastric cancer; (ii) direct gastric cancer rechallenge/readministration studies were prioritized whenever available; (iii) non-gastric cancer rechallenge studies were used only to provide indirect safety or feasibility context; and (iv) mechanistic or biomarker studies were interpreted as biological rationale rather than as evidence of clinical efficacy.

Accordingly, the manuscript should be read as critical narrative synthesis. The purpose is to define evidence gaps, clarify plausible resistance mechanisms, and propose a conceptual framework for future prospective studies, not to claim the certainty level of a systematic review.

## 2 Core mechanisms of immunotherapy resistance in gastric cancer

Immunotherapy resistance in gastric cancer occurs at several biological levels. It can be broadly divided into primary resistance, in which tumors fail to respond from the outset, and acquired resistance, in which disease progresses after an initial response. Key mechanisms include tumor microenvironment remodeling, impaired antigen presentation, T-cell exhaustion, and compensatory upregulation of non-PD-1 immune checkpoints (2, 5).

Evidence-level clarification: The mechanistic sections below are intended to explain biological plausibility and resistance hypotheses. They should not be interpreted as direct clinical evidence that rechallenge improves outcomes in gastric cancer. To maintain balance with the limited clinical evidence, detailed pathway descriptions are framed as candidate mechanisms that require prospective validation and biomarker-stratified clinical correlation.

### 2.1 Distinctive features of primary resistance versus acquired resistance

Primary resistance is linked to the intrinsic properties of tumors. For example, Ras homolog family member A(RHOA) mutations upregulate free fatty acid levels via activating the PI3K-AKT-mTOR pathway, which drives the accumulation of regulatory T cells (Tregs) in TME and fosters an immunosuppressive milieu (6). EBV-negative or diffuse-type gastric cancers often exhibit an immune ignorance state due to inadequate T-cell infiltration and low PD-L1 expression (7, 8). Moreover, soluble legumain (sLGMN) secreted by gastric cancer cells binds to Integrin αvβ3 to activate the PI3K/AKT/mTORC2 pathway, inducing M2 polarization of macrophages and directly mediating primary resistance (9).

Acquired resistance is predominantly driven by adaptive immune escape mechanisms, such as post-treatment defects in antigen presentation machinery, T-cell exhaustion, or compensatory upregulation of checkpoint molecules (2, 9). For example, in resistant patients, elevated plasma IL-4 levels induce upregulation of FcγRIIB via metabolic reprogramming of macrophages (enhanced glycolysis and lactate production), which in turn suppresses CD8^+^ T-cell function (10).

### 2.2 Immunosuppressive mechanisms of the TME

TME can suppress antitumor immunity through several interacting components. To improve readability, these mechanisms are summarized as major categories rather than as an exhaustive pathway catalogue.

Major TME mechanisms include (1): expansion of tumor-associated macrophages, myeloid-derived suppressor cells, and regulatory T cells (2); metabolic remodeling, including Hypoxia-inducible factor 1-alpha (HIF1A)-driven glycolysis, lactate accumulation, and lipid dysregulation (3); stromal exclusion mediated by cancer-associated fibroblasts; and (4) microbiota-associated immune modulation. These mechanisms provide biological rationale for resistance, but they do not by themselves prove clinical benefit from rechallenge (5, 11–22).

### 2.3 Defects in antigen presentation machinery and T cell exhaustion

Antigen presentation impairment: While EBV-positive gastric cancer is enriched with CD8^+^ T cells, some tumors exhibit downregulated expression of Human Leukocyte Antigen (HLA) class I molecules or defects in antigen processing machinery, thereby restricting T cell recognition (2, 7). The MSI-H/dMMR subtype can select for low-immunogenicity clones via immune editing (2).

T Cell Dysfunction: CD8^+^ T cell exhaustion is characterized by sustained high expression of inhibitory receptors (e.g., PD-1, TIM-3, LAG-3) and loss of effector functions (10, 17, 23, 24). Single-cell analyses have revealed functional heterogeneity among T cell subsets within TME, where the exhausted phenotype is directly correlated with therapeutic resistance (25). Furthermore, upregulated LAG-3 expression and downregulated Siglec-7 expression on natural killer (NK) cells impair their cytotoxic activity (23).

### 2.4 Compensatory upregulation mechanisms of checkpoint molecules

Dynamic Alterations in the PD-1/PD-L1 Axis: Following the development of treatment resistance, PD-L1 expression in tumor cells may be downregulated due to attenuated Interferon-gamma (IFN-γ) signaling or sustain high expression via epigenetic remodeling (2, 26). Co-localization of tumor-derived PD-L1 (tPD-L1) with tumor-infiltrating CD8^+^ T cells correlate with favorable prognosis, implying that its expression may reflect immune response activity rather than mere immune evasion (27).

Compensatory Activation of Non-PD-1 Checkpoints: Molecules including Cytotoxic T-Lymphocyte-Associated Protein 4 (CTLA-4), LAG-3, and T-cell immunoreceptor with Ig and ITIM domains (TIGIT) undergo compensatory activation in drug-resistant settings (23). For example, TIM-3 and TIGIT on NK cells are upregulated upon contact with tumor cells (23); Lysyl Oxidase-Like 3 (LOXL3) promotes treatment resistance by modulating the expression of immune checkpoints (28).

V-domain Ig-containing suppressor of T cell activation (VISTA) should also be considered within the non-PD-1 resistance network. GC-specific studies suggest that VISTA is linked mainly to myeloid- and stromal-dominant immune suppression. VISTA-high tumors may contain VISTA-positive monocyte-macrophage populations, exhausted CD8+ T cells, regulatory T cells, M2-like macrophages, and cancer-associated fibroblasts. These observations support VISTA as a candidate resistance mediator and combination target, but they do not establish VISTA-guided rechallenge efficacy. Its role in GC rechallenge remains hypothesis-generating and requires prospective validation (29–31).

**Figure 1:**
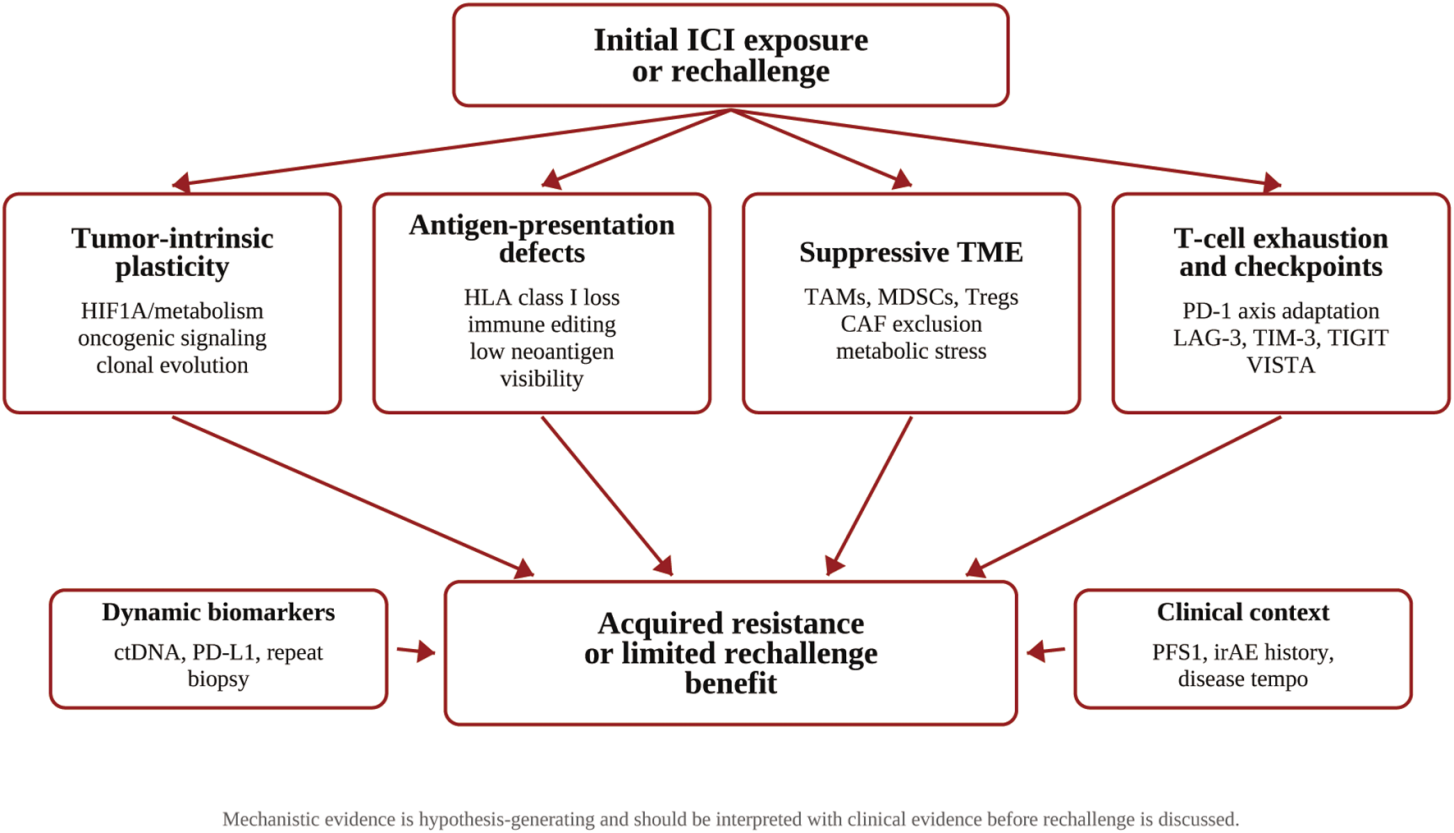
Mechanistic map of ICI resistance and rechallenge in gastric cancer. The diagram summarizes tumor-intrinsic, antigen-presentation, TME, checkpoint, biomarker, and clinical-context mechanisms that may limit rechallenge benefits. It is a conceptual image and does not imply validated clinical efficacy.

Immunosuppressive Molecular Network: Molecules such as Indoleamine 2,3-dioxygenase (IDO), Fc gamma receptor IIB (FcγRIIB), and Lipoma-preferred partner (LPP) contribute to the formation of an inhibitory network. High LPP expression in fibroblasts is associated with reduced infiltration of activated CD4^+^ memory T cells, decreased survival, and treatment resistance (26); Interleukin-4 (IL-4)-induced upregulation of FcγRIIB directly impairs T cell function (10). (**Figure 1**).

## 3 Molecular subtype-guided interpretation of immunotherapy resistance

Molecular subtyping of gastric cancer provides a critical framework for unraveling the mechanisms underlying immunotherapy resistance, as distinct molecular subtypes exhibit unique TME features and immune evasion strategies.

### 3.1 Unique immune features of EBV-positive gastric cancer

EBV-positive gastric cancer often shows an immune-inflamed phenotype, with high PD-L1 expression and dense CD8+ tumor-infiltrating lymphocytes (32–34). PD-L1 overexpression may be driven by CD274 amplification or IFN-gamma-mediated signaling (35). EBV-positive tumors can also express additional checkpoints such as CTLA-4 and VISTA and may show lymphoid-stroma morphology (36–38). These features support biological sensitivity to ICI-based strategies, but antigen-presentation defects and adaptive checkpoint changes may still limit rechallenge benefits.

### 3.2 Immune editing mechanisms in MSI-H/dMMR subtype

MSI-H/dMMR gastric cancer usually has high tumor mutation burden and neoantigen load, but its immune phenotype is heterogeneous. Some tumors contain effectors and exhausted T-cell subsets with high immune activity, whereas others show cold-tumor features, including low CD8+ T-cell infiltration, Treg enrichment, absent tertiary lymphoid structures, and low PD-L1 expression (37, 39). HLA class I defects are frequent and may impair neo-antigen presentation. Tripartite Motif Containing 6(TRIM6) hypermethylation may also weaken Cyclic GMP-AMP Synthase-Stimulator of Interferon Genes(cGAS-STING) signaling and reduce cytotoxic T-cell infiltration. These mechanisms may contribute to resistance and should be viewed as candidate biomarkers rather than validated rechallenge criteria (40, 41).

### 3.3 Dynamic expression and spatial heterogeneity of PD-L1

PD-L1 expression exhibits subtype-specific dynamic patterns and spatial heterogeneity. The PD-L1 positivity rate in EBV^+^ and MSI-H subtypes is significantly higher than in other subtypes (EBV^+^: 81.8%, MSI-H: 73.5% vs. EBV^-^/pMMR: 27.8%) (32, 42). In MSI-H gastric cancer, the number of frameshift mutations correlate positively with PD-L1 expression (43), and PD-L1 expression is strongly associated with CD8-positive Tumor-Infiltrating Lymphocytes (CD8^+^ TIL) density and proximity to tumor cells (33, 42). Spatially, PD-L1 expression is higher at the tumor invasive margin, particularly in EBV^+^ and MSI-H subtypes (4). Notably, 28.4% of EBV^-^/pMMR gastric cancers are PD-L1-negative but show high CD8^+^ TIL infiltration (32), suggesting that single PD-L1 testing may overlook potential responders.

### 3.4 Emerging biomarkers

TMB synergizes with PD-L1 expression to predict immunotherapeutic efficacy in MSI-H gastric cancer (44); the activation status of the IFN-γ signaling pathway, linked to M1-type tumor-associated macrophage(TAM) infiltration, is a prerequisite for PD-L1/PD-1 blockade efficacy (45); IDO1 expression is elevated in EBV^+^ and MSI-H subtypes, potentially mediating resistance (46); TRIM6 methylation status modulates immune infiltration by regulating the cGAS-STING pathway (41). These emerging biomarkers offer novel insights into the deciphering of resistance mechanisms (47–49).

Recent reviews of gastric cancer biomarkers further emphasize that rechallenge decisions should not rely on a single static marker. Instead, PD-L1 CPS, MSI-H/dMMR status, EBV status, TMB, HLA/antigen-presentation defects, IFN-γ signaling, ctDNA dynamics, spatial immune contexture, TAM/cancer-associated fibroblast (CAF) signatures, and emerging proteomic or multi-omics classifiers should be interpreted as complementary layers of evidence (43, 50–52). These markers are clinically promising but remain incompletely validated for the specific rechallenge setting.

Recent biomarker reviews were therefore incorporated as context for patient selection rather than as evidence of rechallenge efficacy. Across this narrative review, PD-L1 Combined Positive Score (CPS), MSI-H/dMMR, EBV status, TMB, DNA Damage Response (DDR)-related alterations, tertiary lymphoid structures, macrophage/fibroblast signatures, and VISTA are presented as candidate biomarkers that may guide future trial design and hypothesis generation, not as validated criteria for routine gastric cancer rechallenge decisions (29–31, 50, 51).

## 4 Clinical evidence for immunotherapy rechallenge in gastric cancer

Because the gastric cancer-specific rechallenge literature remains limited, the evidence discussed below is explicitly separated into: (i) direct gastric cancer rechallenge/readministration evidence; (ii) gastric cancer biomarker or mechanistic evidence that may inform patient selection; and (iii) indirect evidence from other tumor types. Only the first category directly addresses clinical outcomes of rechallenge in gastric cancer. The latter two categories are useful for biological rationale and trial design but should not be interpreted as proof of clinical efficacy in GC. To make the level of evidence explicit, we applied the following interpretive hierarchy throughout the clinical sections (**Table 1**).

**Table 1:** Evidence tiers for interpreting immune checkpoint inhibitor rechallenge in gastric cancer.

Evidence tier	Source type	How it is interpreted in this review
Tier 1: Direct gastric cancer rechallenge evidence	Retrospective GC rechallenge/readministration cohorts or case series	Can suggest feasibility or an efficacy signal but remains low or very-low certainty because of sample size, selection bias, heterogeneous regimens, and lack of randomized controls.
Tier 2: Gastric cancer translational/biomarker evidence	GC biomarker cohorts, single-cell/spatial studies, VISTA/TME analyses, ctDNA or molecular subtype studies	Supports patient-selection hypotheses and trial stratification but does not prove rechallenge benefit.
Tier 3: Non-gastric cancer rechallenge evidence	Lung cancer, melanoma, esophageal cancer, or mixed-tumor rechallenge/reintroduction series	Provides indirect context for feasibility and safety only; efficacy cannot be extrapolated to GC without validation.
Tier 4: Preclinical/mechanistic evidence	Cellular, animal, organoid, omics, and pathway studies	Defines plausible resistance biology and rational combinations; it is hypothesis-generating rather than clinically confirmatory.

This hierarchy was used to soften conclusion-level statements and to separate evidence-based observations from hypotheses.

### 4.1 Clinical efficacy signals and evidence limitations of ICI rechallenge

Current clinical evidence for immune checkpoint inhibitor (ICI) rechallenge in gastric cancer remains limited and should be interpreted cautiously. The available gastric cancer-specific data are dominated by retrospective, non-randomized studies, including one 60-patient retrospective cohort and very small nivolumab readministration or rechallenge series. In the largest GC-specific retrospective cohort, 60 patients received anti-PD-1 rechallenge after prior anti-PD-1-based therapy. The median progression-free survival after rechallenging (PFS2) was 2.9 months, the objective response rate (ORR) was 16.7%, and the disease control rate (DCR) was 55.0%. Rechallenge with the same PD-1 antibody was associated with longer median PFS2 than switching to a different PD-1 antibody (3.5 vs. 1.9 months; *p* = 0.007). PD-L1 positivity and PFS1 ≥ 6 months appeared to be favorable factors, whereas peritoneal metastasis was associated with poorer PFS2 (53). A smaller nivolumab readministration series included eight patients with advanced gastric cancer, among whom six were rechallenged after disease progression and two were reintroduced to nivolumab after immune-related pneumonitis. Among the six rechallenge cases, the median PFS after rechallenge was 1.8 months and two patients achieved stable disease; among the two reintroduction cases, no recurrent pneumonitis was reported (54). Earlier small gastric cancer series similarly reported limited activity, with short PFS and no consistent objective responses (55).

Together, these observations suggest that ICI rechallenge or readministration may be feasible and may provide disease control in selected patients, but they should be regarded as exploratory rather than confirmatory. The strength of evidence is limited by small sample sizes, retrospective design, treatment heterogeneity, selection bias, inconsistent definitions of “rechallenge,” “readministration,” “reintroduction,” and “continued ICI beyond progression,” as well as incomplete endpoint and biomarker standardization (53–55). Patients selected for rechallenge often have favorable clinical features, such as good performance status, slower disease tempo, prior ICI tolerance, oligoprogression, or longer initial benefit, making it difficult to distinguish a true treatment effect from favorable patient selection. Confounding is also substantial because ICI rechallenge is frequently combined with chemotherapy, targeted therapy, anti-angiogenic agents, or local treatment, which limits attribution of benefit to PD-1/PD-L1 blockade alone (56–58). In addition, biomarker variables, including PD-L1 CPS, MSI/dMMR status, EBV status, tumor mutation burden, ctDNA dynamics, prior duration of response, and resistance pattern, have not been consistently collected or prospectively stratified (59–61). Therefore, although PD-L1 positivity, longer initial ICI benefit, absence of peritoneal disease, lower tumor burden, and good performance status are clinically plausible selection factors, they remain exploratory and should not be considered validated criteria for routine rechallenge.

Accordingly, VISTA-focused studies, and recent biomarker reviews are discussed in this review as gastric cancer-specific supportive or hypothesis-generating evidence rather than as direct clinical proof of rechallenge efficacy (30, 62). These studies may help explain why some tumors remain immune-sensitive or develop targetable adaptive resistance, but they do not establish ICI rechallenge as a standard strategy for second-line or later treatment in gastric cancer. Cross-cancer rechallenge data should also be interpreted as indirect evidence and used only to inform safety considerations, terminology, and future trial design, rather than as evidence that gastric cancer patients will benefit from rechallenge. Overall, the strength of evidence for ICI rechallenge in gastric cancer is low to very low and rechallenge should be considered only for carefully selected patients after multidisciplinary assessment, preferably within a clinical trial or registry, rather than as an established standard of care (**Table 2**).

**Table 2:** Summary of key clinical, translational, and indirect evidence related to ICI rechallenge or readministration in gastric cancer.

Evidence category	Representative evidence	Population/design	Main finding or implication	Level of support
Direct GC rechallenge	Zhang et al. clinical analysis of anti-PD-1 rechallenge (53)	Advanced gastric cancer; retrospective cohort; n=60	Median PFS2: 2.9 months; ORR 16.7%; DCR 55.0%; same PD-1 antibody associated with longer PFS2 than switching; peritoneal metastasis was an adverse factor.	Low; retrospective; hypothesis-supporting, not definitive
Direct GC readministration/rechallenge	Kodama/Narita et al. nivolumab readministration case series (54)	Advanced gastric cancer; retrospective case series; n=8, including 6 rechallenge after progression and 2 reintroduction after pneumonitis	In rechallenge cases, median PFS 1.8 months; two patients achieved SD. Feasible in selected patients, but activity was limited and evidence very small.	Very low; case-series evidence
Direct GC rechallenge	Tsuji et al. nivolumab rechallenge (55)	Advanced gastric cancer; small retrospective series	Short PFS and limited objective responses; supports a cautious interpretation.	Very low
Direct GC biomarker evidence	Xu et al. composite biomarker panel (63)	Advanced gastric cancer; cohort biomarker analysis	Suggests that lymphocyte count, ATM mutation, TMB, and PD-L1 may stratify ICI response; not a formal rechallenge trial.	Low to moderate for biomarker association; indirect for rechallenge
GC multi-omics/immune-sensitivity evidence	Li et al., 2025 (126)	Initially unresectable gastric cancer; immunotherapy-based conversion treatment with multi-omics analysis	Provides GC-specific context for immune sensitivity/resistance and potential selection; does not directly establish rechallenge efficacy.	Hypothesis-generating
GC mechanistic resistance	Zhang et al., 2024 IL-4/IL-4R-FcγRIIB axis (10)	Mechanistic study of macrophage-mediated immunotherapy resistance	Supports myeloid/macrophage-mediated resistance as a rational combination target; indirect for rechallenge.	Preclinical/mechanistic
GC VISTA biology	Luo et al. VISTA-high GC (29); Cao et al. VISTA blockade/TAM reprogramming (30); Lee et al. VISTA in PCC-GC (31)	Translational and retrospective biomarker studies in gastric cancer	Supports VISTA as a myeloid/stromal checkpoint and candidate resistance mediator; not direct clinical rechallenge evidence.	Translational; hypothesis-generating
Indirect rechallenge data	Esophageal cancer, lung cancer, melanoma rechallenge/reintroduction series (16, 69, 127)	Non-gastric cancer cohorts	Inform feasibility and safety concepts but cannot be directly extrapolated to gastric cancer efficacy.	Indirect

### 4.2 Response heterogeneity across patients with distinct resistance subtypes

Response heterogeneity is a central reason why gastric cancer rechallenge cannot be recommended uniformly. In the available clinical data, longer benefit from initial ICI exposure, PD-L1 positivity, absence of extensive peritoneal disease, better performance status, and lower tumor burden appear to be associated with more favorable outcomes, but these signals are retrospective and not validated as prospective selection criteria (53–55, 63). Mechanistic studies suggest that antigen-presentation defects, T-cell exhaustion, macrophage-dominant immunosuppression, VISTA expression, and other non-PD-1 resistance nodes may shape rechallenge response. These factors should therefore be presented as candidate stratification variables for future trials, not as established clinical decision rules.

### 4.3 Synergistic mechanisms of combination therapy strategies

Combination strategies may theoretically overcome acquired resistance by targeting complementary pathways such as VEGF-mediated immunosuppression, macrophage polarization, CAF-driven exclusion, VISTA-positive myeloid suppression, or compensatory checkpoints. However, most data supporting these approaches are mechanistic, early-phase, or derived from non-rechallenge settings. Therefore, in the rechallenge context, combination therapy should be described as a rational investigational strategy rather than a proven standard of care (64, 65).

For clinical interpretation, the most defensible recommendation is cautious patient selection: rechallenge may be considered only in highly selected patients with prior meaningful ICI benefit, preserved performance status, limited disease burden or oligoprogression, manageable prior immune-related Adverse Events(irAEs), and a plausible biomarker rationale. Whenever feasible, enrollment in prospective trials or biomarker-driven studies should be prioritized. (These recommendations are grounded in hypothesis-generation, and future prospective trials are essential to substantiate these findings).

### 4.4 Safety management: irAEs and rechallenge timing

IrAEs are a critical limiting factor for rechallenge or reintroduction. In the gastric cancer-specific retrospective cohort, grade 1–2 and grade 3–4 adverse events occurred in 83.3% and 35.0% of patients, respectively, indicating that toxicity is clinically relevant even when considered manageable (53). In the nivolumab readministration case series, two patients restarted nivolumab after grade 2 pneumonitis without recurrent pneumonitis, but this observation is based on only two patients and should not be generalized (54). Rechallenge after serious irAEs should therefore require careful multidisciplinary risk assessment, documentation of irAE resolution, and close monitoring.

Because gastric cancer-specific safety data for ICI rechallenge are sparse, safety conclusions should be framed with the same caution as efficacy conclusions. In this review, gastric cancer-specific observations are separated from indirect evidence derived from other tumors. The available GC data show that adverse events are common during rechallenge-based treatment, but they do not provide sufficiently granular information to define organ-specific recurrence risks, optimal steroid washout intervals, or safe rechallenge criteria after severe irAEs. Therefore, safety decisions should be individualized rather than protocolized solely from the current GC literature (66, 67).

Before ICI re-exposure, the clinical scenario should be clarified. Reintroduction after toxicity, continuation beyond progression, and true rechallenge after progression are biologically and clinically different. For patients with prior irAEs, clinicians should document the affected organ, toxicity grade, treatment required, time to resolution, residual impairment, steroid or immunosuppressive use, and available non-ICI options. Rechallenge is more defensible after fully resolved low-grade toxicity. It is much less appropriate after life-threatening or unresolved toxicity, especially severe pneumonitis, myocarditis, severe neurologic toxicity, or other grade 4 events, unless the expected benefit is exceptional and multidisciplinary consensus is reached (68–70).

Several safety checkpoints should be documented before rechallenging. First, clinicians should define the scenario: reintroduction after toxicity, continuation beyond progression, or true rechallenge after progression. Second, they should record the previous irAE grade, affected organ, treatment required, recovery status, and current steroid or immunosuppressive exposure. Third, they should assess performance status, tumor burden, tempo of progression, alternative therapies, overlapping toxicities, and the monitoring plan. During the first 8–12 weeks after rechallenging, close surveillance is advisable because recurrent or new irAEs may occur early and may be difficult to distinguish from chemotherapy toxicity or tumor-related symptoms. These recommendations are practical risk-management principles, not validated GC-specific rules (71–75).

## 5 Precision decision-making framework

The proposed three-dimensional decision matrix is not a validated treatment algorithm. It is a structured clinical reasoning and trial-design tool built on three axes. Axis 1 captures molecular-immune subtype, including MSI-H/dMMR, EBV status, PD-L1 CPS, TMB, and antigen-presentation integrity. Axis 2 captures dynamic biomarkers and response kinetics, including ctDNA trend, PD-L1 change, immune contexture, and prior depth or duration of ICI benefit. Axis 3 captures resistance and safety pattern, including oligoprogression versus rapid systemic progression, peritoneal disease burden, performance status, and prior irAE phenotype. No single axis is sufficient to justify rechallenge (**Table 3**).

**Table 3:** operationalizes the three axes of the matrix and links them to practical decision points.

Axis	Assessment	Supports rechallenge	Argues against	Decision implication
Axis 1: molecular-immune subtype	MSI/dMMR, EBV, PD-L1 CPS, TMB, HLA/APM, immune phenotype	MSI-H/dMMR or EBV+, PD-L1+, intact antigen presentation, inflamed TME	MSS/EBV-, HLA loss, immune-desert or CAF/TAM exclusion, no biomarker rationale	Provides rationale; not sufficient alone.
Axis 2: dynamic biomarkers and kinetics	ctDNA, radiologic tempo, PFS1, prior response, repeat biopsy when feasible	Long initial benefit, durable response, low/falling ctDNA, oligoprogression	Primary progression, rapid ctDNA rise, short PFS1, diffuse progression	Helps identify residual immune sensitivity.
Axis 3: resistance and safety pattern	Progression pattern, peritoneal burden, ECOG, organ function, prior irAE	Limited burden, local therapy feasible, resolved low-grade irAE, good PS, monitoring plan	Peritoneal carcinomatosis, poor PS, severe/unresolved irAE, no monitoring plan	Risk and trajectory are co-primary determinants.

The practical outputs of the matrix are intentionally conservative. Trial enrollment or registry capture is preferred. Selected rechallenge may be discussed only when all three axes support persistent immune sensitivity and acceptable safety. Rational combinations should be tested prospectively. Patients with primary resistance, rapid systemic progression, severe unresolved irAEs, or no biomarker rationale should generally receive non-ICI alternatives or clinical trials rather than empiric rechallenge.

The matrix should therefore be documented as a multidisciplinary discussion rather than a binary yes/no rule. Its current role is to standardize how hypotheses are generated and how future studies may stratify patients.

### 5.1 Rechallenge stratification pathway based on molecular subtyping

Molecular subtyping by immunohistochemistry and *in situ* hybridization can identify clinically relevant gastric cancer subgroups. MSI-H/dMMR and EBV-positive tumors may provide stronger biological rationale for ICI-based strategies. By contrast, genomic stable, epithelial-mesenchymal transition-like, or immune-excluded tumors often have poorer prognosis and may require non-ICI or rational combination approaches. Because subtype alone is insufficient, rechallenge decisions should integrate molecular features with response kinetics, tumor burden, and safety profile (76–79).

### 5.2 Regimen adjustment guided by dynamic biomarker monitoring

Dynamic biomarker monitoring is critical for optimizing rechallenge strategies. Long non-coding RNAs (lncRNAs) in body fluids exhibit high specificity and serve as dynamic biomarkers for predicting chemotherapy response and assessing prognosis (80). A 15-gene signature panel constructed based on TME- and metabolism-related genes can distinguish high-risk stromal phenotypes from low-risk epithelial phenotypes (81). The mesenchymal (MP) subtype is characterized by increased stromal components, M2 macrophage infiltration, and dysregulated metabolic pathways; it is resistant to immunotherapy but sensitive to IGF-1R/PI3K-mTOR inhibitors (81). Circulating tumor DNA (ctDNA) analysis combined with dynamic PD-L1 expression monitoring enables real-time tracking of tumor clonal evolution (82–84).

### 5.3 Management of special populations

Special populations require individualized assessment. In elderly patients, simplified immunohistochemistry-based molecular subtyping may provide a practical and low-cost prognostic tool (85, 86). In patients with prior irAEs, rechallenge decisions should focus on the previous toxicity phenotype, recovery status, inflammatory context, and organ-specific risk. Potential markers such as macrophage infiltration, Interleukin-34 (IL-34), Macrophage Colony-Stimulating Factor(M-CSF), and PD-L1 spatial heterogeneity remain exploratory and should not replace clinical judgment (87–89).

### 5.4 Artificial intelligence-assisted efficacy prediction models

AI-assisted models may improve response prediction by integrating molecular, histologic, imaging, and microenvironmental data. Examples include DNA-stratification classifiers, programmed-cell-death pathway models, and cancer-associated fibroblast scores (90–92). However, these tools are not validated for GC rechallenge. The MODIN model was developed in breast cancer and should be cited only as a conceptual example, not as a standard model for gastric cancer (93).

Artificial intelligence, organoid models, and multi-omics technologies may support future precision rechallenge strategies. AI models could integrate molecular data, imaging, ctDNA kinetics, PD-L1 expression, MSI-H/dMMR status, and clinical trajectories to help distinguish pseudoprogression from true progression. Multi-omics analyses may further define resistance phenotypes and identify patients for biomarker-driven trials (94–97). These applications remain exploratory and require prospective validation before routine use.

Patient-derived organoids may help model individual tumor biology and drug sensitivity. Immune-organoid co-culture systems could be used to test PD-1/PD-L1 rechallenge or combination strategies ex vivo (98, 99). However, standardized protocols and clinically validated predictive thresholds are still lacking (99, 100).

Despite the important translational value of the above technologies, their clinical implementation still faces numerous challenges, including the lack of unified standardized protocols, high difficulty in integrating multi-omics data with clinical information, and the requirement for consistent validation of predictive performance across different molecular subtypes of gastric cancer. Therefore, AI and organoid technologies are still in the exploratory and preliminary application stage, and their widespread clinical application in immunotherapy rechallenge for gastric cancer needs to be fully validated through large-scale, prospective, multi-center clinical trials.

### 5.5 Stepwise clinical application and illustrative scenarios

Stepwise application guidance: Step 1, define the scenario as reintroduction after toxicity, continuation beyond progression, or true rechallenge after progression. Step 2, document prior ICI benefit, regimen, treatment-free interval, and irAE history. Step 3, classify progression as oligoprogression, indolent systemic progression, or rapid disseminated progression. Step 4, reassess biomarkers when feasible, including MSI/dMMR, EBV, PD-L1 CPS, TMB, ctDNA kinetics, and evidence of antigen-presentation or TME-mediated resistance. Step 5, discuss the case in a multidisciplinary setting and prioritize trial or registry enrollment. Step 6, if rechallenge is chosen, predefine the regimen, monitoring schedule, interruption criteria, and alternative plan.

Hypothetical scenario A: A patient with EBV-positive or MSI-H/dMMR gastric cancer achieved a durable partial response to first-line ICI-based therapy, discontinued or changed treatment after prolonged disease control, and later developed isolated nodal or hepatic oligoprogression with preserved performance status and no severe prior irAE. This patient represents a biologically plausible but still investigational rechallenge candidate, ideally within a prospective trial or with local therapy plus carefully monitored ICI re-exposure.

Hypothetical scenario B: A patient with MSS/EBV-negative diffuse-type gastric cancer had primary progression within two to three months of PD-1 exposure, rising ctDNA, extensive peritoneal carcinomatosis, declining performance status, and no favorable biomarker signal. In this setting, empiric PD-1/PD-L1 rechallenge is not supported by current evidence; standard chemotherapy, targeted therapy when indicated, best supportive care, or clinical trial options should be prioritized.

Hypothetical scenario C: A patient previously discontinued ICI because of grade 2 colitis or pneumonitis that fully resolved without ongoing immunosuppression and subsequently developed progression after a meaningful treatment-free interval. Reintroduction or rechallenge may be discussed only after organ-specific recovery is confirmed, alternative therapies are reviewed, and early monitoring for recurrent irAE is planned. This scenario is distinct from rechallenge after severe, life-threatening, or unresolved toxicity, where re-exposure should generally be avoided.

These scenarios are illustrative and should not be interpreted as recommendations for routine clinical use. They are included to show how the proposed matrix may support structured discussion while preserving appropriate uncertainty.

## 6 Discussion and outlook

### 6.1 Integration of core findings: a multidimensional synergistic network of rechallenge resistance

This review critically synthesizes resistance mechanisms and clinical observations relevant to ICI rechallenge in gastric cancer. The central conclusion is deliberately conservative: direct GC-specific clinical evidence remains limited, while most mechanistic and biomarker data are hypothesis-generating. Therefore, the proposed molecular subtype-guided framework should be interpreted as a research and clinical reasoning tool, not as a validated treatment algorithm.

### 6.2 Molecular subtyping-guided rechallenge decision-making framework

Molecular subtyping helps explain resistance heterogeneity but rechallenge decisions should move beyond static subtype labels. EBV-positive tumors may be immune-inflamed, yet some develop HLA class I loss or other antigen-presentation defects. MSI-H/dMMR tumors are also heterogeneous and may show cold-tumor features that limit immune response (32, 35, 37, 39, 40). Therefore, subtype information should be integrated with dynamic biomarkers, response kinetics, immune contexture, and safety profile.

The temporal dimension of dynamic PD-L1 expression is increasingly recognized. Spatially, PD-L1 expression is higher at the tumor invasive margin, particularly in EBV^+^ and MSI-H subtypes (101); temporally, ctDNA monitoring during treatment enables real-time tracking of tumor-immune interaction dynamics (82, 84). Notably, 28.4% of EBV^-^/pMMR gastric cancers are PD-L1-negative but exhibit high CD8^+^ TIL infiltration (32), indicating that single PD-L1 testing may overlook potential responders.

Based on these insights, a three-dimensional decision matrix integrating molecular subtype, dynamic biomarkers, and resistance pattern may be useful for trial design and individualized discussion. Its clinical utility remains unvalidated. The revised framework is shown in **Table 3**; it should be applied only as a structured discussion tool, not as a validated treatment pathway.

1. EBV-positive or MSI-H/dMMR tumors may have stronger biological rationale for ICI-based strategies, but rechallenge should still require assessment of antigen-presentation integrity, immune contexture, prior depth/duration of response, and available standard options;
2. Patients with longer prior ICI benefit, PD-L1 positivity, controlled tumor burden, and oligoprogression may be considered more plausible candidates for rechallenge or ICI continuation with local therapy, but this remains a low-evidence, individualized approach;
3. Patients with rapid primary progression, extensive peritoneal disease, poor performance status, or clear non-PD-1 resistance biology should generally be directed toward standard chemotherapy, targeted therapy, clinical trials, or rational combination approaches rather than empiric PD-1 rechallenge.

### 6.3 Optimization of clinical rechallenge strategies

Clinical optimization should avoid overstating the current evidence. At present, gastric cancer rechallenge data support feasibility in selected patients, not broad efficacy. The practical recommendation is to distinguish ICI reintroduction after toxicity, ICI continuation beyond progression, and true rechallenge after progression, because these scenarios differ biologically and clinically (55, 102, 103). Future studies should standardize these definitions and report outcomes separately.

Potential candidates for prospective rechallenge studies may include patients with durable initial response or stable disease on ICI, long treatment-free interval, oligoprogression amenable to local therapy, favorable biomarker profile, and absence of uncontrolled peritoneal carcinomatosis. Conversely, patients with primary resistance, rapid systemic progression, or severe unresolved irAEs are unlikely to be appropriate candidates for empiric rechallenge.

Management of synergistic toxicity in combination therapy is a critical limitation of rechallenge. Patients with a history of irAEs have a higher risk of severe toxicity recurrence, especially those with prior grade ≥3 irAEs, requiring rigorous benefit-risk assessment (68, 69, 104). Patients with low-grade checkpoint inhibitor pneumonitis, favorable performance status, and low Interleukin-6(IL-6)/C-reactive Protein (CRP) levels at rechallenge have lower recurrence risk (105), which can inform patient selection.

From a clinical decision-making perspective, safety should be treated as a co-primary consideration rather than a secondary issue. A reasonable rechallenge discussion should require three simultaneous conditions: a plausible likelihood of antitumor benefit, acceptable risk based on the prior irAE phenotype and recovery status, and a predefined monitoring and interruption plan (106–109). If any of these conditions are absent, standard chemotherapy, targeted therapy, local therapy for oligoprogression, or clinical trial enrollment without empiric PD-1/PD-L1 re-exposure may be safer alternatives.

### 6.4 Current controversies and research gaps

Several unresolved issues limit clinical translation:

Lack of standardized dynamic biomarker monitoring: PD-L1 expression exhibits significant spatiotemporal heterogeneity (50, 51), and ctDNA monitoring thresholds and cross-platform consistency are unestablished (110);

Evidence gap in rechallenge timing and dosing: Lung cancer studies suggest that patients with at least 12 months of initial immunotherapy benefit may have better rechallenge outcomes (111), but GC-specific evidence is scarce. Dose adjustment after prior irAEs is still based largely on clinical experience rather than prospective data (69, 70);

Limited predictive capacity for synergistic toxicity: There is a lack of reliable models to predict irAE recurrence or toxicity overlap in immune-targeted combinations (112, 113);

Underdeveloped gastric cancer-specific safety evidence: most practical safety considerations for ICI rechallenge are still extrapolated from melanoma, lung cancer, esophageal cancer, or mixed-tumor cohorts. Gastric cancer patients frequently receive chemotherapy, anti-angiogenic agents, nutritional support, and multiple-line treatment, which may increase attribution difficulty and clinical vulnerability (114, 115). Future GC-specific rechallenge studies should prospectively capture prior irAE type and grade, steroid exposure, time to irAE resolution, rechallenge interval, recurrence of the same irAE, occurrence of new irAEs, treatment discontinuation, hospitalization, and immune-related mortality.

Insufficient gastric cancer-specific evidence and limited critical certainty: Most rechallenge knowledge remains extrapolated from other cancers or derived from retrospective GC cohorts and small case series. These studies are limited by small sample sizes, lack of randomized control groups, heterogeneous rechallenge definitions, treatment-selection bias, confounding by combination therapy, and incomplete biomarker annotation. The available direct GC data suggest feasibility and occasional disease control, but they do not establish rechallenge as a standard treatment. Prospective, biomarker-stratified trials with uniform definitions and prespecified endpoints are required before guideline-level recommendations can be made (53–55).

### 6.5 Future research directions and clinical translation prospects

Based on this integrated analysis, we propose the following priority research directions:

Clonal evolution tracking for optimal rechallenge timing: Single-cell sequencing and spatial omics can dissect the evolutionary trajectory of resistant clones (110), while liquid biopsy enables real-time monitoring. Key questions include: What is the optimal rechallenge window? Which molecular signals (e.g., *de novo* driver mutations, antigen-presenting gene silencing) indicate rechallenge failure risk?

Patient-derived organoids (PDOs) for individualized prediction: PDOs retain primary tumor genomic heterogeneity and drug responsiveness (116–118), enabling resistance model construction and high-throughput screening of reversal strategies. Future directions include: establishing a large-scale gastric cancer PDO biobank covering diverse subtypes and resistance stages (118); integrating PDO drug sensitivity testing with multi-omics to build individualized prediction models; developing immune-organoid co-culture systems to simulate TME immune cell crosstalk for rechallenge evaluation (25).

Epigenetic modulator combinations: Histone modifications (H3K4 demethylation, H3K27 acetylation) (119–121), dynamic DNA methylation (122), and non-coding RNA regulation (123, 124) play core roles in resistance. Histone Deacetylase (HDAC) inhibitors, Protein Arginine Methyltransferase (PRMT) inhibitors, and DNA demethylating agents can reverse immune gene silencing (121, 125), but optimal combination timing (simultaneous vs. sequential), dose optimization, and toxicity management require further exploration.

AI-integrated multi-omics prediction models: Deep learning integrating genomic, transcriptomic, proteomic, and radiomic data can overcome single-biomarker limitations. Current progress includes a 10-protein biomarker model with AUC = 0.959 (102) and a CAF scoring system outperforming Tumor Immune Dysfunction and Exclusion (TIDE) and Immunophenotype Score (IPS) in immunotherapy response prediction (75). Prospective studies are needed to validate AI model clinical utility, and explainable AI should be explored to reveal underlying biological mechanisms.

## 7 Conclusion

Clinical decision-making for immunotherapy rechallenge in gastric cancer should remain conservative and evidence qualified. Direct GC-specific evidence is limited to retrospective cohorts and small case series. Reported outcomes are modest and heterogeneous. Therefore, rechallenge is not an established standard of care. It may be considered only for carefully selected patients after multidisciplinary assessment and, ideally, within prospective or biomarker-driven studies. Study-level limitations, including sample size, retrospective design, selection bias, confounding treatment effects, and incomplete biomarker stratification, should be explicitly acknowledged.

Mechanistic and biomarker findings—including molecular subtype, PD-L1, MSI-H/dMMR, EBV status, TMB, ctDNA dynamics, antigen-presentation defects, macrophage/CAF signatures, and VISTA—provide a valuable framework for hypothesis generation and trial design, but they are not yet validated criteria for routine rechallenge decisions.

Safety is a central determinant of rechallenge feasibility. The current gastric cancer literature provides limited direct evidence regarding irAEs recurrence after rechallenging, and therefore practical safety recommendations must rely on cautious integration of GC-specific observations, indirect rechallenge data, and general irAE management principles. Rechallenge should be avoided or approached only exceptionally after severe, life-threatening, or unresolved irAEs, whereas carefully monitored re-exposure may be considered in selected patients with resolved low-grade toxicity and a strong clinical rationale.

Future work should prioritize prospective gastric cancer-specific rechallenge trials with standardized definitions, clear separation of rechallenge versus reintroduction, mandatory biomarker collection, and preplanned toxicity monitoring. Only through such evidence can the field move from empiric rechallenge toward genuinely precision-guided rechallenge.

## Glossary

ICIs: Immune checkpoint inhibitors
GC: gastric cancer
TME: tumor microenvironment
Tregs: regulatory T cell
EBV: Epstein-Barr virus
sLGMN: soluble legumain
NK: natural killer
VISTA: V-domain Ig-containing suppressor of T cell activation
lncRNAs: Long non-coding RNAs
ctDNA: Circulating tumor DNA
PDOs: Patient-derived organoids
CAF: cancer-associated fibroblast
LAG-3: Lymphocyte-activation gene 3
TIM-3: T-cell immunoglobulin and mucin-domain containing-3
TIGIT: T-cell immunoreceptor with Ig and ITIM domains
MSI-H: Microsatellite instability-high
dMMR: Deficient mismatch repair
PD-L1: Programmed Death-Ligand 1
PD-1: Programmed Death-1
RHOA: Ras Homolog Family Member A
HIF1A: Hypoxia-inducible factor 1-alpha
HLA: Human Leukocyte Antigen
IFN-γ: Interferon-gamma
CTLA-4: Cytotoxic T-Lymphocyte-Associated Protein 4
TIGIT: T-cell immunoreceptor with Ig and ITIM domains
FcγRIIB: Fc gamma receptor IIB
LPP: Lipoma-preferred partner
IDO: Indoleamine 2,3-dioxygenase
IL-4: Interleukin-4
cGAS-STING: Cyclic GMP-AMP Synthase-Stimulator of Interferon Genes
TRIM6: Tripartite Motif Containing 6
pMMR: Mismatch Repair Proficient
CD8^+^ TIL: CD8-positive Tumor-Infiltrating Lymphocytes
TAM: tumor-associated macrophage
CPS: Combined Positive Score
DDR: DNA Damage Response
PFS: Progression-free survival
irAEs: immune-related Adverse Events
IL-34: Interleukin-34
M-CSF: Macrophage Colony-Stimulating Factor
AI: Artificial Intelligence
CRP: C-reactive Protein
IL-6: Interleukin-6
PDOs: Patient-derived organoids
PRMT: Protein Arginine Methyltransferase
HDAC: Histone Deacetylase
TIDE: Tumor Immune Dysfunction and Exclusion
IPS: Immunophenotype Score
ORR: objective response rate
DCR: disease control rate
LOXL3: Lysyl Oxidase-Like 3
